# Serum Metabolomic Profiles in Neonatal Mice following Oral Brominated Flame Retardant Exposures to Hexabromocyclododecane (HBCD) Alpha, Gamma, and Commercial Mixture

**DOI:** 10.1289/EHP242

**Published:** 2016-11-04

**Authors:** David T. Szabo, Wimal Pathmasiri, Susan Sumner, Linda S. Birnbaum

**Affiliations:** 1 National Human Environmental Exposure Research Laboratory, U.S. Environmental Protection Agency (EPA), Research Triangle Park, North Carolina, USA; 2 Curriculum in Toxicology, University of North Carolina–Chapel Hill, Chapel Hill, North Carolina, USA; 3 Discovery Sciences, Research Triangle Institute International, Research Triangle Park, North Carolina, USA; 4 National Institute of Environmental Health Sciences, and; 5 National Toxicology Program, National Institutes of Health (NIH), Department of Health and Human Services, Research Triangle Park, North Carolina, USA

## Abstract

**Background:**

Hexabromocyclododecane (HBCD) is a high production volume brominated flame retardant added to building insulation foams, electronics, and textiles. HBCD is a commercial mixture (CM-HBCD) composed of three main stereoisomers: α-HBCD (10%), β-HBCD (10%), and γ-HBCD (80%). A shift from the dominant stereoisomer γ-HBCD to α-HBCD is detected in humans and wildlife.

**Objectives:**

Considering CM-HBCD has been implicated in neurodevelopment and endocrine disruption, with expected metabolism perturbations, we performed metabolomics on mice serum obtained during a window-of-developmental neurotoxicity to draw correlations between early-life exposures and developmental outcomes and to predict health risks.

**Methods:**

Six female C57BL/6 mice at postnatal day (PND) 10 were administered a single gavage dose of α-, γ-, or CM-HBCD at 3, 10, and 30 mg/kg. Nuclear magnetic resonance metabolomics was used to analyze 60 μL serum aliquots of blood collected 4 days post-oral exposure.

**Results:**

Infantile mice exposed to α-, γ-, or CM-HBCD demonstrated differences in endogenous metabolites by treatment and dose groups, including metabolites involved in glycolysis, gluconeogenesis, lipid metabolism, citric acid cycle, and neurodevelopment. Ketone bodies, 3-hydroxybutyrate, and acetoacetate, were nonstatistically elevated, when compared with mean control levels, in all treatment and dose groups, while glucose, pyruvate, and alanine varied. Acetoacetate was significantly increased in the 10 mg/kg α-HBCD and was nonsignificantly decreased with CM-HBCD. A third ketone body, acetone, was significantly lower in the 30 mg/kg α-HBCD group with significant increases in pyruvate at the same treatment and dose group. Metabolites significant in differentiating treatment and dose groups were also identified, including decreases in amino acids glutamate (excitatory neurotransmitter in learning and memory) and phenylalanine (neurotransmitter precursor) after α-HBCD and γ-HBCD exposure, respectively.

**Conclusions:**

We demonstrated that 4 days following a single neonatal oral exposure to α-, γ-, and CM-HBCD resulted in different serum metabolomic profiles, indicating stereoisomer- and mixture-specific effects and possible mechanisms of action.

**Citation:**

Szabo DT, Pathmasiri W, Sumner S, Birnbaum LS. 2017. Serum metabolomic profiles in neonatal mice following oral brominated flame retardant exposures to hexabromocyclododecane (HBCD) alpha, gamma, and commercial mixture. Environ Health Perspect 125:651–659; http://dx.doi.org/10.1289/EHP242

## Introduction

1,2,5,6,9,10-hexabromocyclododecane (HBCD) is a brominated flame retardant (BFR) produced globally as a commercial-mixture (CM-HBCD). CM-HBCD, which is added to consumer products, enters the environment during production and leaches from goods in use or disposal (EC 2008). Food and dust ingestion and inhalation are considered relevant human exposure routes in the United States, Canada, European Union (EU), China, New Zealand, and Africa (Ali et al. 2012; Kalachova et al. 2012; Shoeib et al. 2012; Asante et al. 2013; Ni and Zeng 2013; Eljarrat et al. 2014; Fromme et al. 2014). CM-HBCD is lipophilic (log K_ow_ 5.6), resistant to environmental degradation, has half-lives of 51, 1,440, and 5,760 hr in air, water, and sediment, respectively, transports over long distances, and bioaccumulates in human and animal tissue (EC 2008).

CM-HBCD was added to the 2013 Stockholm Convention list for global elimination following PBT (persistence, bioaccumulation, and toxicity) evaluation with a 5-year exemption for building insulation (see http://chm.pops.int/default.aspx). In 2014, the U.S. Environmental Protection Agency (EPA) nominated CM-HBCD to be added to the Toxic Release Inventory (U.S. EPA 2014b), and to the U.S. EPA’s Design for the Environment Alternatives Assessments (U.S. EPA 2014a). In the EU, CM-HBCD is listed as a substance of very high concern under Annex XIV of REACH (Regulation for Registration, Evaluation, Authorisation and Restriction of Chemicals); after 21 August 2015, only approved applications may use the chemical (ECHA 2009). Currently, under the Commission for Environmental Cooperation (CEC), Canada, Mexico, and the United States are evaluating the presence and migration of CM-HBCD from consumer products (CEC 2015). CM-HBCD is currently on the U.S. EPA Integrated Risk Information System (IRIS) program agenda, but an anticipated date for completion has not yet been determined (U.S. EPA 2015).

CM-HBCD public health concerns focus on adverse effects to infants and young children, as it has been detected in human fetal livers (Rawn et al. 2014), and experimental evidence suggests that it can impact thyroid hormone (TH), energy and lipid metabolism, and neurodevelopment. Young mice (6–20 weeks) that were exposed weekly to 700 μg/kg or 35 μg/kg CM-HBCD in a high-fat diet experience weight gain and metabolic dysfunction (disrupting lipid and glucose homeostasis), possibly accelerating obesity progression (Yanagisawa et al. 2014). A two-generation Crl:CD(SD) rat (Ema et al. 2008) and pregnant Sprague-Dawley rat study (Saegusa et al. 2009) found no observed adverse effect level (NOAEL)at 150 ppm and 100 ppm CM-HBCD doses, respectively, while higher levels of CM-HBCD led to disruption of TH levels. Learning and memory and other developmental neurotoxicity (DNT) end points were observed in 3-month-old mice after a single oral exposure of either 0.9 mg HBCD/kg or 13.5 mg HBCD/kg to neonatal NMRI mice (Eriksson et al. 2006). Pregnant Long-Evans rats, gavaged gestation day 1 to parturition with 3, 10, or 30 mg/kg HBCD, produced offspring with long-term behavioral impairments (Miller-Rhodes et al. 2014). PND22 Balb/c mice exposed 28 days to fish-based diets spiked with CM-HBCD had changes in neural transcriptomic profiles (calcium signaling) and proteomic profiles (excitotoxicity) (Rasinger et al. 2014), possibly inhibiting sarcoplasmic-endoplasmic reticulum Ca^2+^ ATPase (Al-Mousa and Michelangeli 2014). Sixty-two children from randomly selected women who were part of the prospective Groningen infant COMPARE study noted to have detectable serum HBCD concentrations were found to have changes in coordination, verbal skills, and total intelligence (Roze et al. 2009). In a cross-sectional study of 515 Belgian adolescents 14–17 years old, serum HBCD concentrations were not significantly correlated with neurobehavioral test scores or TH levels (Kiciński et al. 2012).

CM-HBCD stereoisomers are gamma (γ-HBCD; 75–89%), alpha (α-HBCD; 10–13%), and beta (β-HBCD; 1–12%) (Heeb et al. 2005). γ-HBCD is highest in environmental matrices, while α-HBCD dominates humans and wildlife, demonstrating the importance of stereoisomer-specific biomonitoring and toxicity studies (Covaci et al. 2006). HBCD stereoisomer studies demonstrate differences in water solubility, lipophilicity, structure, toxicokinetics, and metabolic pathways (Szabo et al. 2010, 2011a). γ-HBCD is rapidly metabolized and eliminated (terminal half-life ~ 1–4 days) with limited bioaccumulation, and undergoes *in vivo* stereoisomerization to β- and α-HBCD (Szabo et al. 2010). In contrast, α-HBCD’s terminal half-life is ~ 17 days, and it bioaccumulates, with lipophilic-driven tissue distribution, and no *in vivo* stereoisomerization (Szabo et al. 2011a). The toxicokinetic differences account for stereoisomer shifts between γ-HBCD and α-HBCD. Szabo et al. (2011b) demonstrated that infantile mice exposed at PND10 (α-HBCD or γ-HBCD) had 10–25% increased body-burden over adults (PND60). Hakk et al. (2012) identified unique metabolites via radiochemical detection in the urine and feces of α-HBCD and γ-HBCD-treated mice, further suggesting stereoisomers differ biologically and chemically. However, *in vivo* mammalian stereoisomer-specific toxicity studies are limited.

This study uses a non-targeted metabolomics approach to predict potential DNT hazards from exposure to a commercial chemical mixture and individual stereoisomers. Metabolomics enables the assessment of changes in levels of low-molecular weight endogenous compounds after chemical exposure. The goals of this study include *a*) develop a neonatal serum screening method to identify endogenous responses following CM-HBCD exposure; *b*) compare profiles between CM-HBCD, α-HBCD, and γ-HBCD; and *c*) determine metabolites associated with treatment groups and that have been associated with DNT in other studies.

## Methods and Materials

### Chemicals

1,2,5,6,9,10-hexabromocyclododecane (CAS#3194-55-6) is CM-HBCD purchased from Sigma-Aldrich (St. Louis, MO). Stereoisomer separation and thermal conversion were previously described (Szabo et al. 2010, 2011a). Other chemicals utilized were purchased from Sigma-Aldrich (St. Louis, MO) at the highest purity level available.

### Dosing Solutions

Doses were selected based on published DNT (Eriksson et al. 2006) and toxicokinetics (Szabo et al. 2010, 2011a, 2011b), and stereoisomer percentage in CM-HBCD, α-HBCD, γ-HBCD, and CM-HBCD were individually mixed with corn oil and toluene and evaporated under vacuum; control solutions were generated using the same procedure. Mice were gavaged with 3, 10, or 30 mg/kg α-HBCD, 3 and 30 mg/kg γ-HBCD, and 30 mg/kg CM-HBCD. The doses selected were designed to determine individual stereoisomer contribution when compared to the commercial mixture [e.g., ~ 10% alpha (3 mg/kg) and 90% gamma (30 mg/kg) metabolic profiles to the 30 mg/kg CM-HBCD].

### Animals and Treatment

PND10 mice given a single oral exposure of 0.9 mg/kg or 13.5 mg/kg HBCD displayed significant differences in spontaneous behavior at 3 months of age, and mice given the higher dose also had signs of impaired memory and learning (Eriksson et al. 2006) during a window of developmental neurotoxicity; therefore, this time point was selected for this metabolomic study. Mouse dams (*n* = 7) and female C57BL/6 pups (PND9, ~ 7 g) were purchased from Charles River Breeding Laboratories (Raleigh, NC), housed in an Association for Assessment and Accreditation of Laboratory Animal Care (AAALAC)-accredited facility, under the U.S. EPA’s National Health and Environmental Effects Research Laboratory (NHEERL) and Institutional Animal Care and Use Committee (IACUC) approval. Dams and six female pups per litter were acclimated (24 hr) in shoebox cages. PND10 female pups were gavaged with a single dose of vehicle control, α-HBCD, γ-HBCD, or CM-HBCD, at 10 mL/kg. Rodents were maintained on a 12-hr light/dark cycle, ambient temperature (22°C), and relative humidity (56 ± 5%). Rodents were provided Purina 5001 Rodent Chow (Ralston Purina, St. Louis, MO) and tap water *ad libitum*. Three to six pups per treatment (one pup per litter) were sacrificed (decapitation) 4 days following exposure, individually weighed, and blood harvested. Protocols involving animal use have been approved by an appropriate institutional committee, and animals have been treated humanely and with regard for alleviation of suffering.

### Sample Preparation

We chose to measure blood metabolites on PND14 based on tissue concentrations measured at same time point (Szabo et al. 2010, 2011a, 2011b), and allowance of ample time to capture metabolite formation. Serum was prepared and stored at –80°C until analyzed. Aliquot of 60 μL of serum was mixed with 80 μL of solution containing 5 mM formate, 0.2% NaN_3_ in D_2_O, and 260 μL of saline (0.9% NaCl in D_2_O); 400 μL of solution was transferred into 5 mm NMR tube.

### Instrument and Data Acquisition

^1^H NMR spectra were acquired on Bruker Advance III 950 MHz instrument (located at DHMRI, Kannapolis, NC) using a CPMG pulse sequence (Beckonert et al. 2007) with water suppression (cpmgpr1d) during relaxation, a 2-sec relaxation delay, and 256 scans. Spectra were acquired at 25°C, with 32 k data points, and zero-filled and Fourier Transformed. Spectra were phase- and baseline-corrected manually, and referenced to formate (8.44 ppm). The quality of NMR spectra was assessed for NMR line shape and width, signal to noise levels, alignment of identified markers. These are standard measures that ensure the quality of NMR data for further analysis. NMR peaks in the spectra had symmetric lorentzian line shapes with good signal to noise ratio and appropriate line width ~ 1–1.5 Hz, demonstrated by the line width of the internal standard peak, formate. The chemical shift (position of the NMR peaks in the spectrum) is sensitive to factors such as pH and ionic strength in the sample. Characteristic peaks in serum samples such as glucose and lactate were used for alignment across the samples. All raw and processed data, metadata, and experimental procedures are available at the NIH common fund metabolomics workbench (http://www.metabolomicsworkbench.com/).

### Data Preprocessing

NMR data were preprocessed using traditional binning and quantitative approaches (Sumner et al. 2010; Pathmasiri et al. 2012). Binning was performed by automated integration with a 0.04 ppm bin width over the spectral window (excluding water suppression regions and formate signal), and bins were normalized to total integral of each of the spectrum. Serum metabolites and concentrations were library matched with Chenomx NMR Suite 5.1 Professional software (Edmonton, Alberta) using the formate internal standard for relative integration. This software contains internal library adjustments for increments in chemical shift based on pH, and relaxation time of signal (Weljie et al. 2006). The following advantages of this method include: *a*) small increments in pH result in portions of metabolite signals aligning with different bins, while deconvolution circumvents issue, and *b*) multiple signals within separate bins.

NMR spectroscopy is a quantitative method and the area under the peak is directly proportional to the number of atoms (^1^H described in the current analysis) underlying the peak. The relative concentration of the metabolite(s) can be determined by using an internal standard with a known concentration and the integrals of the metabolite and internal standard peaks using the formula C_M_ = C_IS_ × (I_M_/I_IS_)(N_IS_/N_M_), where C_M_ = concentration of metabolite, C_IS_ = concentration of internal standard, I_M_ = integral of metabolite peak, I_IS_ = integral of the internal standard, N_M_ = number of ^1^H atoms of the metabolite peak, and N_IS_ = number of ^1^H atoms of the internal standard peak. The NMR solutions used in these samples contained 1 mM formate, hence, enabling the relative quantitation of metabolites in the sample with respect to the formate internal standard. The Chenomx NMR library used for relative concentration determination contains a quantitative metabolite library where this formula is pre-built into software such that the concentration of metabolites can be directly obtained when the information about the internal standard and its concentration is given (Weljie et al. 2006). For subsequent data reduction from library matching, metabolite concentration was normalized to formate.

### Data Reduction and Visualization

NMR data capture (metabolite ID and concentration or bin region and integral value) were transferred to SIMCA-P+ 12.0 software (Umetrics; Umeå, Sweden) for reduction and visualization. Normalized binned NMR data were Pareto scaled by dividing the integral of each bin by the reciprocal of the square root of the standard deviation for the bin and centered prior to multivariate analysis. Principal component analysis (PCA) and partial least squares projection to latent structures discriminate analysis (PLS-DA) were conducted using SIMCA-P+ 12.0 for binned and concentration data. These pattern recognition methods are commonly used to analyze high dimensional multicollinear data such as metabolomics data (Trygg et al. 2007; Eriksson et al. 2013). Loadings, variable influence on projections (VIP), and contribution plots were examined to determine bins or metabolites that best define group separation, which are commonly used multivariate statistical analysis approaches (Eriksson et al. 2013). The VIP statistic summarizes the importance of the bin in differentiating the phenotypic groups. The subset of bins or metabolites that had a VIP ≥ 1.0 with a jack-knife confidence interval that did not include 0 were determined to be important for differentiating the study groups. All models used a 7-fold cross-validation to assess the predictive variation of the model (Q2) (Eriksson et al. 2013). In addition, metabolite concentrations were compared between treatment and dose groups to controls using Mann–Whitney *U* test.

## Results

**Figure 1:**
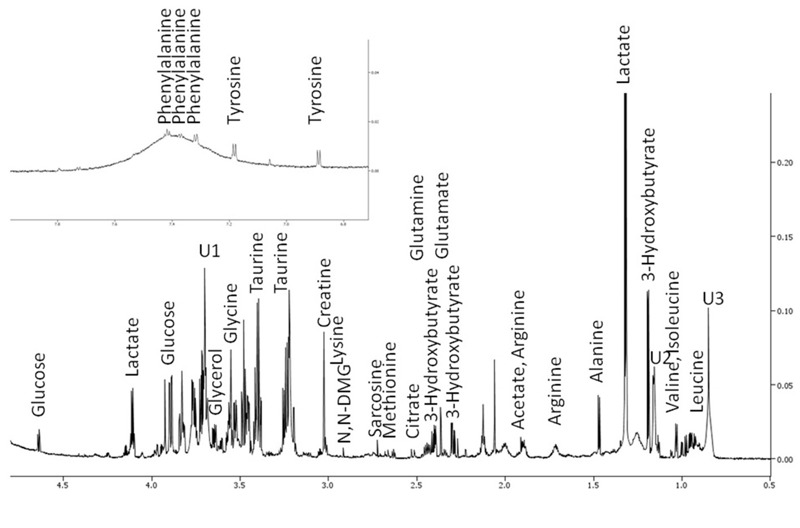
950 MHz ^1^H NMR spectrum of metabolites in a representative 60-μL mouse serum preparation. Signals for metabolites at higher concentration are labeled.

This study used 60 μL serum aliquots and enabled the library matching of 40 endogenous metabolites for the treatment and dose groups for samples collected on the fourth day following a single oral exposure at PND10 (see Table S1). No changes in body weight were observed between treated and control mice (data not shown). NMR signals assigned via Chenomx library matching identified essential and nonessential amino acids, alcohols, ketones, fatty-acid by-products, and sugars (Figure 1). Classes of compounds detected by the NMR method included amino acids (alanine, asparagine, arginine, cysteine, glycine, glutamate, glutamine, homoserine, isoleucine, leucine, lysine, methionine, phenylalanine, sarcosine, serine, threonine, tyrosine, taurine, valine); alcohols (methanol and glycerol); ketones and fatty acid by-products (acetoacetate, acetate, 3-hydroxybutyrate, acetone, pyruvate, isobutyrate); sugar (glucose); other small molecule intermediates (o-phosphocholine, choline, n,n-dimethylglycine, citrate, creatine, lactate, methylhistidine, succinate, myo-inositol, dimethylamine, methylsuccinate, and 2-hydroxyisobutyrate).

Significant differences in the metabolite-response levels and direction of change across treatment and dose groups are summarized in Table 1, and mean values of all metabolites are listed according to treatment group in Table S1. Phenylalanine exhibited decreases, 36% and 42% (*p* = 0.02 and 0.03, respectively) from controls after exposure to γ-HBCD at 3 mg/kg and 30 mg/kg, respectively. Glutamate exhibited dose-dependent decreases of 14%, 17%, and 28% (*p* = 0.37, 0.05, and 0.05, respectively) in 3 mg/kg, 10 mg/kg, and 30 mg/kg α-HBCD groups. Arginine was significantly decreased 30% and 48% (*p* = 0.05 and 0.02, respectively) in 3 mg/kg from γ- and α-HBCD, respectively. O-phosphocholine and choline increased 29% (*p* = 0.05) and decreased 43% (*p* = 0.007) in 10 mg/kg and 30 mg/kg groups, respectively, after α-HBCD exposure. Ketone bodies, acetoacetate and acetone, increased 40% (*p* = 0.02) at 10 mg/kg and decreased 28% (*p* = 0.03) at 30 mg/kg with α-HBCD exposure. Glycerol and taurine increased after CM-HBCD exposure by 18% (*p* = 0.02) and 11% (*p* = 0.01), respectively. Compared with controls, serum metabolites display mean values either consistently elevated (> 0) across all treatment and dose groups (3-hydroxybutyrate, creatine, valine, leucine) or lowered (< 0) across all treatment and dose groups (hemoserine, alanine, arginine), while all others display treatment- and dose-specific responses (see Table S1).

**Table 1:** Metabolites that were significantly higher or lower than controls in 60 μL serum aliquots from at least one group of HBCD-exposed mice.

Metabolite	α-HBCD			γ-HBCD		Commercial HBCD
	3 mg/kg *n* = 3	10 mg/kg *n* = 3	30 mg/kg *n* = 6	3 mg/kg *n* = 3	30 mg/kg *n* = 6	30 mg/kg *n* = 6
Increased
Acetoacetate	1.05 ± 0.07	1.40 ± 0.14*	1.19 ± 0.35	1.05 ± 0.43	1.06 ± 0.05	–1.08 ± 0.02
Glycerol	–1.11 ± 0.24	1.07 ± 0.41	–1.03 ± 0.09	1.2 ± 0.23	1.10 ± 0.23	1.18 ± 0.09*
O_phosphocholine	–1.02 ± 0.10	1.29 ± 0.18*	–1.01 ± 0.28	–1.01 ± 0.28	–1.09 ± 0.07	1.22 ± 0.14
Taurine	1.04 ± 0.40	1.08 ± 0.12	–1.00 ± 0.001	1.3 ± 0.83	1.07 ± 0.25	1.11 ± 0.64*
Pyruvate	1.10 ± 0.22	1.13 ± 0.36	1.39 ± 0.12*	1.08 ± 0.50	1.12 ± 0.27	1.00 ± 0.35
Decreased
Acetone	–1.02 ± 0.33	1.16 ± 0.5	–1.28 ± 0.5*	1.04 ± 0.01	1.00 ± 0.01	1.02 ± 0.33
Arginine	–1.30 ± 0.018*	–1.05 ± 0.20	–1.02 ± 0.36	–1.48 ± 0.95*	–1.02 ± 0.29	–1.09 ± 0.44
Choline	–1.06 ± 0.14	1.04 ± 0.13	–1.43 ± 0.01*	1.04 ± 0.17	–1.12 ± 0.29	1.14 ± 0.47
Glutamate	–1.14 ± 0.21	–1.17 ± 0.06*	–1.28 ± 0.06*	1.07 ± 0.18	–1.18 ± 0.14	1.05 ± 0.19
Phenylalanine	1.00 ± 0.30	–1.03 ± 0.04	–1.15 ± 0.13	–1.36 ± 0.55*	–1.42 ± 0.16*	–1.21 ± 0.04
Note: Values represent difference ± standard deviation. Mean values for all metabolites are presented in Table S1. *Significantly different from control, *p* < 0.05.						

**Figure 2:**
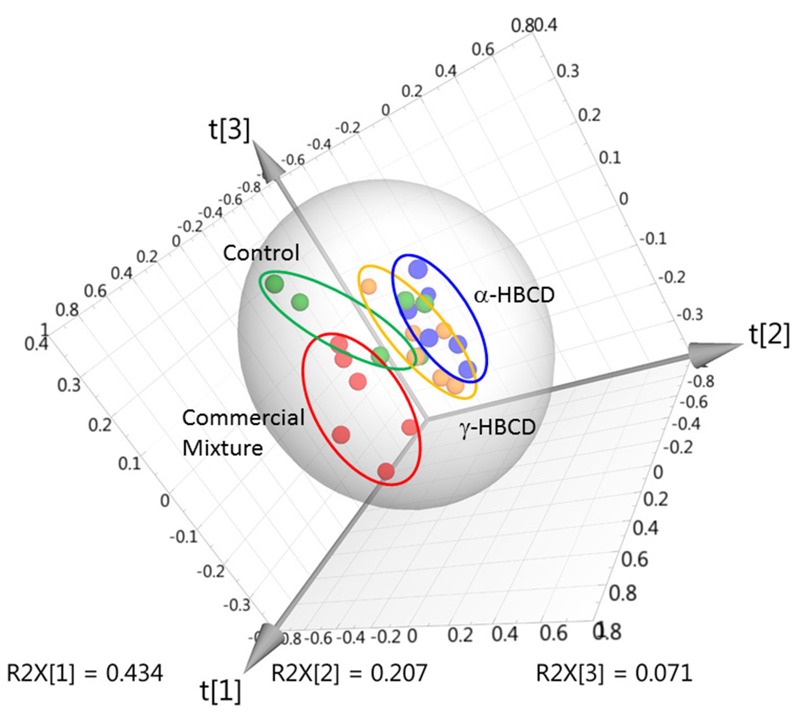
Multivariate analysis (PLS-DA score plot) of bin data obtained for serum from individual (*n *= 6/treatment group) mice exposed to vehicle controls (green), or 30 mg/kg α-HBCD (blue), γ-HBCD (orange), or CM-HBCD (red) [R2X = 0.713; R2Y = 0.379; Q2 = 0.0307].

The PLS-DA of binned data for vehicle control (green), α-HBCD (blue), γ-HBCD (orange), and CM-HBCD (red) at 30-mg/kg group is displayed in Figure 2. The samples derived from mice exposed to either α-HBCD or γ-HBCD cluster and traject on one side of the vehicle control group, while samples from CM-HBCD cluster and traject on the other side of the vehicle control group. There is more variation between treatment and dose groups than within treatment groups. This qualitative metric is supported by observing the patterns of differentiation for treatment and dose groups indicate increased similarity in metabolic profiles between mice exposed to same treatment and dose groups, compared with other groups.

**Figure 3:**
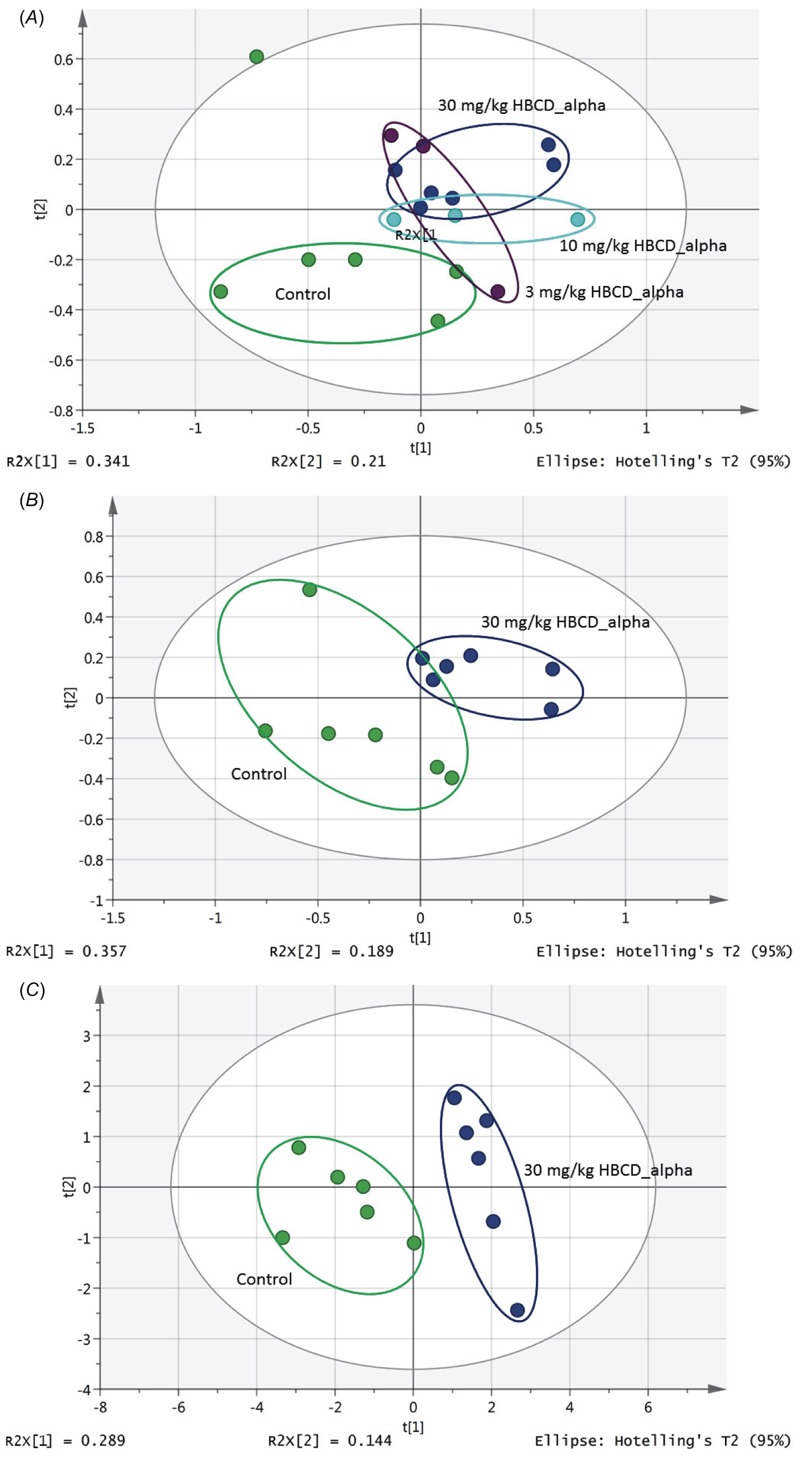
Score plot of PLS-DA analysis of metabolite concentration data for serum samples from mice administered (*A*) 0, 3, 10, and 30 mg/kg doses of α-HBCD, indicating separation between control (green) and dose groups [R2X = 0.664; R2Y = 0.693; Q2 = –0.11]; (*B*) vehicle control or 30 mg/kg α-HBCD showing clear separation of study groups (green, left, control; blue, right, α-HBCD) [R2X = 0.76; R2Y = 0.814; Q2 = 0.13]; (*C*) vehicle controls or 30 mg/kg α-HBCD showing improvement of separation of dose group from control group (green, left, control; blue, right, α-HBCD) [R2X = 0.504; R2Y = 0.915; Q2 = 0.538] was achieved using subset of metabolites that best defined groups (VIP ≥ 1.0 with 95% confidence interval that did not include 0). See Table 1 for numbers of mice per group.

PLS-DA analysis of the concentration data differentiates treatment- and dose-groups. Using α-HBCD profiles as an example, the 3 mg/kg, 10 mg/kg, and 30 mg/kg groups, and vehicle controls are presented in a 2-dimensional PLS-DA scores plot (Figure 3A). PLS-DA was conducted using data for controls and 30 mg/kg (Figure 3B), and examination of loadings plots, variable importance plots, and contribution plots (not shown) enabled selection of a metabolite subset (see “Methods and Materials”) that most contributed to group separation. Using only this metabolite subset, separation of α-HBCD from controls in PLS-DA (Figure 3C) improved, compared with the PLS-DA plot in Figure 3B, confirming that metabolite profiles are indeed distinct between groups. The process was repeated using γ-HBCD and CM-HBCD (control vs. each dose) to derive a list of distinct metabolites best distinguishing each group from other groups (Table 2). Although differentiation was achieved between all treatment and dose groups, PLS-DA analysis of the high-dose group concentration data (30 mg/kg) versus controls provided best separation (data not shown).

**Table 2:** Metabolites important to the differentiation of treatment and control groups, using PLD-DA analysis (VIP ≥ 1.0 with a jack-knife confidence interval that did not include 0).

Exposure	Increased relative to control	Decreased relative to control
α-HBCD 3 mg/kg	3-hydroxybutyrate Acetoacetate Creatine Leucine Methionine O-Phosphocholine Taurine Tyrosine Valine	Alanine Arginine Glutamate Lactate Pyruvate Serine
α-HBCD 10 mg/kg	3-hydroxybutyrate Acetoacetate Glutamine Methionine O-Phosphocholine Taurine	Lactate Methanol N,N-Dimethylglycine Phenylalanine Pyruvate Glutamate
α-HBCD 30 mg/kg	2-hydroxyisobutyrate 3-hydroxybutyrate Acetoacetate Glutamine Leucine Taurine Valine	Acetate Alanine Choline Citrate Glutamate Lactate Methanol Phenylalanine Pyruvate
γ-HBCD 3 mg/kg	3-hydroxybutyrate Citrate Creatine Glycerol Glycine Serine Taurine	Alanine Asparagine Cysteine Lactate Methanol Phenylalanine Pyruvate
γ-HBCD 30 mg/kg	2-hydroxyisobutyrate 3-hydroxybutyrate Asparagine Citrate Creatine Glucose Glycerol Glycine Taurine	Choline Glutamate Lactate Methanol Phenylalanine Pyruvate Serine Threonine Tyrosine
CM-HBCD 30 mg/kg	3-hydroxybutyrate Asparagine Citrate Creatine Glycerol Lactate Methionine O-Phosphocholine Taurine Valine	Alanine Arginine Glucose Methanol Phenylalanine
Note: VIP, variable influence on projections.		

**Figure 4:**
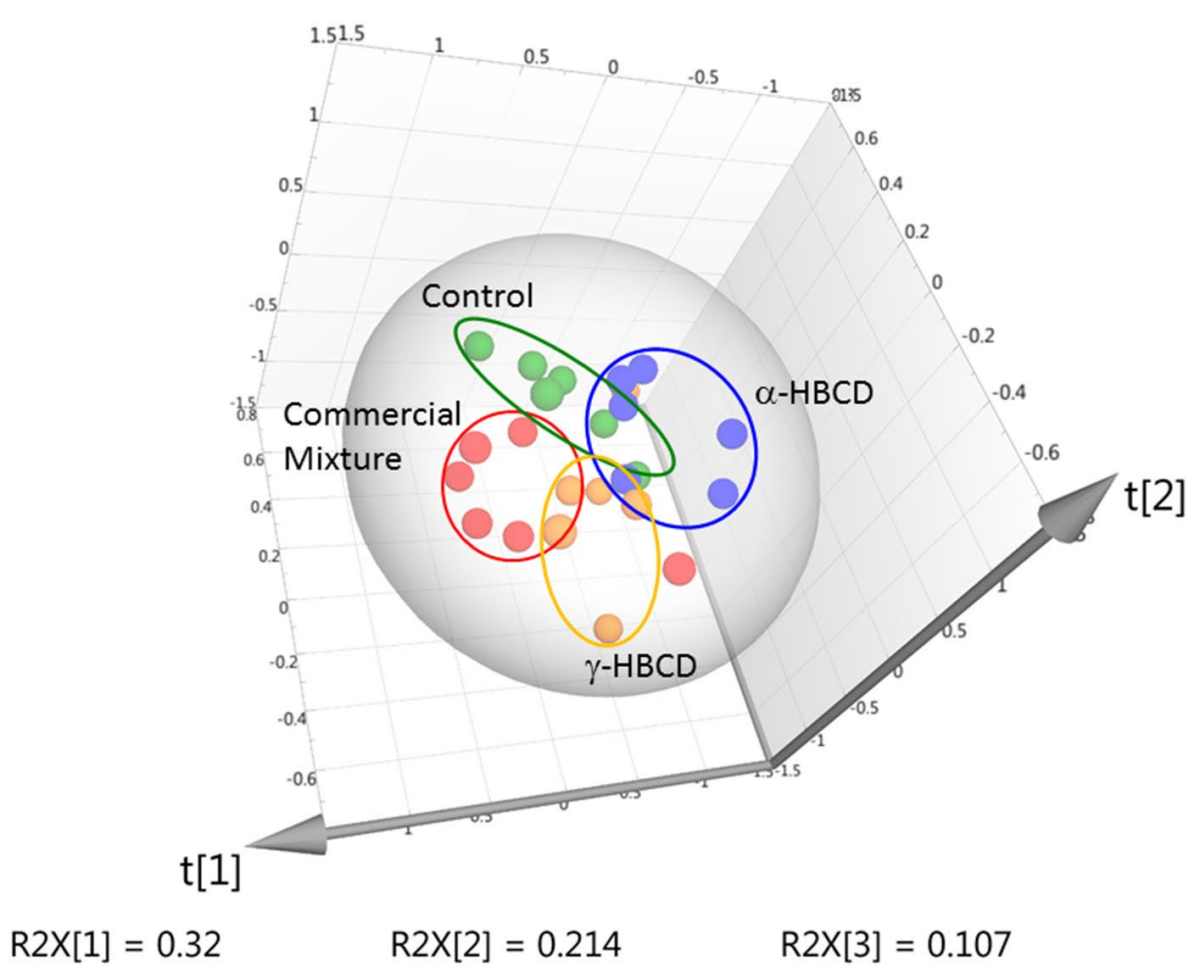
PLS-DA plot of metabolite concentrations derived from serum samples from mice administered vehicle control (green), or high dose (30 mg/kg) CM-HBCD (red), α-HBCD (blue), or γ-HBCD (orange) [R2X = 0.642; R2Y = 0.345; Q2 = –0.186] (*n* = 6 for all groups).

In both PLS-DA and NMR analyses, the CM-HBCD, α-HBCD, and γ-HBCD groups differentiated, representing exposure-specific responses. The CM-HBCD metabolomics pattern is more similar to γ-HBCD, than to α-HBCD (Figure 4). The differences between and among study groups was improved using concentration data, compared with using the binning results.

## Discussion

This study aids the promise of metabolomics in predicting developmental outcomes related to early-life exposures to mixtures and individual constituents of CM-HBCD. The impact of α-HBCD, γ-HBCD, and CM-HBCD on serum biochemical profiles 4 days following acute exposure at PND10 was investigated. Metabolite profiles for the HBCD treatment groups were distinct with a few metabolites in common. Based on prior knowledge, selected metabolites differentiated the treatment and dose groups mapped to different biochemical pathways involving energy and amino acid metabolism that were identified, specifically, those metabolites involved with lipid metabolism and tricarboxylic acid (TCA) cycle.

After CM-HBCD exposure, serum glycerol levels increased, an indication of accelerated lipolysis (lipid metabolism and breakdown) (Herrera and Amusquivar 2000). Acetone, a ketone body, was significantly lower in mice treated with 30 mg/kg α-HBCD compared with controls and may be a result of reduced conversion of acetoacetate to acetone further resulting in reduced conversion of acetone to pyruvate (statistically significant at same treatment and dose group), lactate, and acetate (non-statistically significant trend) (Table 1). Other indicators of accelerated lipolysis were seen with non-statistical increased trends in two other ketone bodies, 3-hydroxybutyrate (all treatments; α-HBCD, γ-HBCD, or CM-HBCD) and acetoacetate (α-HBCD treatment only) (see Table S1), further supporting the possible connection between HBCD exposures and accelerated lipolysis. CM-HBCD effects on hepatic glucose and energy metabolism have been previously described in the literature (Cantón et al. 2008; van der Ven et al. 2006). Rat hepatocytes treated 28 days with CM-HBCD altered gene expression that is involved in glucose metabolism (Cantón et al. 2008). Liver glucose and lipid metabolism disturbances are classic hallmarks of obesity (Muoio and Newgard 2006) and chemical exposures during vulnerable windows of development may cause and contribute to this obesity (La Merrill and Birnbaum 2011). In a rat study, CM-HBCD exposure has been shown to suppresses serum TH levels (van der Ven et al. 2006). TH has been suspected to be involved in energy metabolism (Yanagisawa et al. 2014) leading to decreased energy metabolism and weight gain.

**Figure 5:**
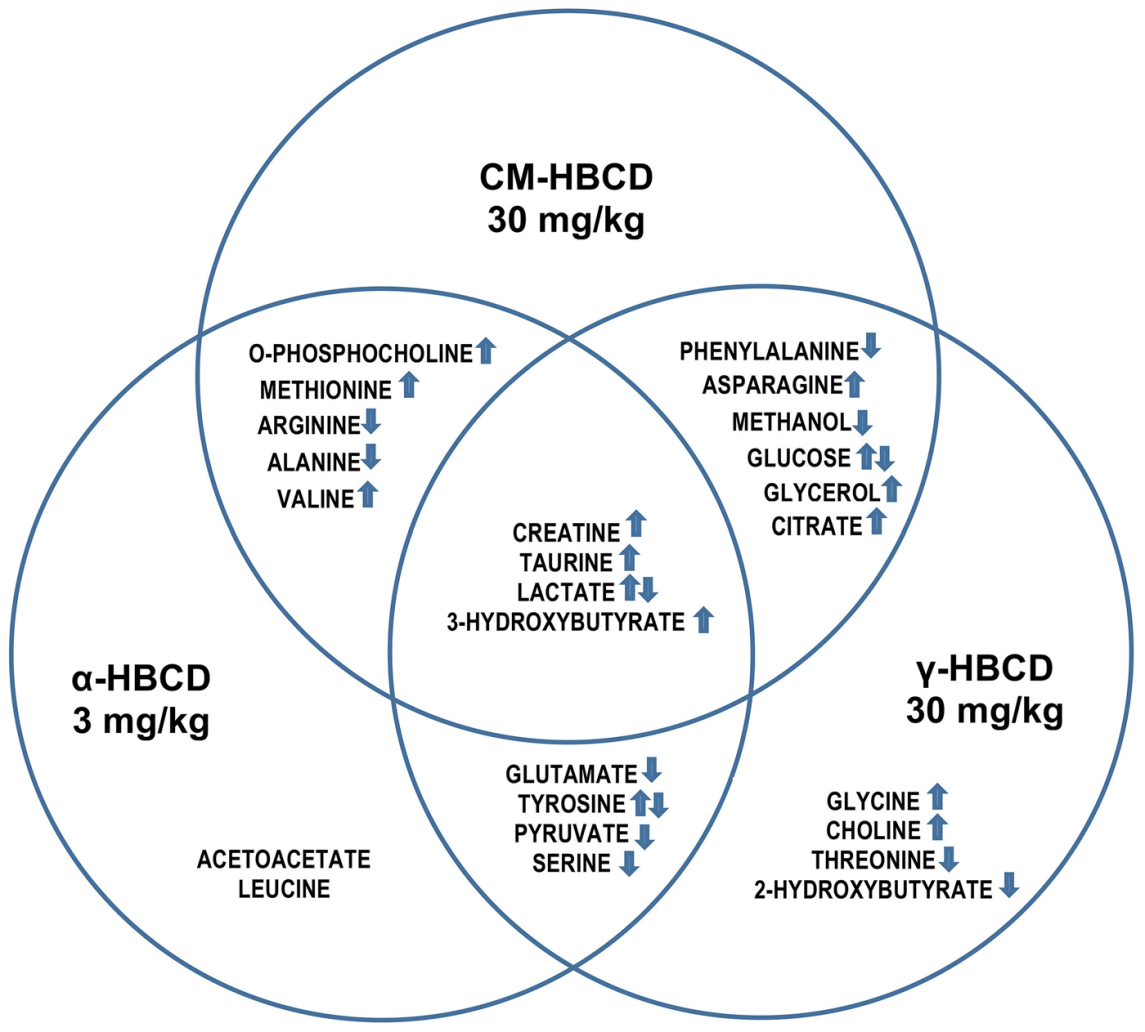
Endogenous serum metabolite increase and decrease after administration of CM-HBCD, α-HBCD, or γ-HBCD at 30 mg/kg, 3 mg/kg and 30 mg/kg, respectively, measured 4 days after an acute dose in infant mice. Contributions of each stereoisomer to the mixture at CM-HBCD relevant ratio ~ 10%:90%::3 mg/kg:30 mg/kg::α-HBCD:γ-HBCD. Arrows indicate metabolites that were consistently increased (↑) or decreased (↓), or that had mixed responses (↑↓).

Elevated non-statistically significant trends in serum 3-hydroxybutyrate, creatine, and taurine response were a similar biomarker fingerprint profile in α-HBCD, γ-HBCD, and CM-HBCD (Figure 5). The specific biomarker profiles (3-hydroxybutyrate, creatine, and taurine), if observed and validated in subsequent longer duration and alternate species studies, could find use in prediction of HBCD exposure and DNT. 3-Hydroxybutyrate, a ketone body used as energy by the brain when blood glucose is low, is essential during brain development, and increased levels are needed to nourish the brain during times of fasting or energy dysregulation (McKenna et al. 2015; Hawdon 1999). Creatine is important in the CNS, facilitating neurotransmitter release, membrane potential maintenance, Ca^2+^ homeostasis, and ion gradient restoration (Stockler et al. 2007). Creatine deficiency syndromes have common clinical manifestations, including cognitive dysfunction with mental retardation, poor language skills, and autism spectrum disorders (Mercimek-Mahmutoglu and Salomons 2015; Tarnopolsky and Beal 2001). The observed increase in creatine and increase in o-phosphocholine may be related to effects on the SAM pathway (Brosnan et al. 2011). Taurine crosses the blood–brain barrier and is implicated in inhibitory neurotransmission, long-term potentiation (LTP) in striatum and hippocampus, membrane stabilization, adipose tissue regulation, possible obesity prevention, calcium homeostasis, protection against glutamate excitotoxicity, and epileptic seizure prevention in humans (Wu and Prentice 2010). The presence of 3-hydroxybutyrate, creatine, and taurine metabolites in all HBCD treatment groups may be driven by α-HBCD, considering γ-HBCD’s biological stereoisomerization to α-HBCD (Szabo et al. 2010) and α-HBCD biological stability (Szabo et al. 2011a).

This metabolomics study also revealed stereoisomer-specific responses, with differences between isomers and their mixture. For α-HBCD, glutamate significantly decreased following mid- and high-dose exposures. The contribution of glutamate to synaptic transmission, plasticity, and development is well established (Morello and Partanen 2015). For γ-HBCD, phenylalanine decreased significantly in both γ-HBCD dose groups. Phenylalanine is an essential amino acid highly concentrated in brain and blood, and the brain uses phenylalanine to produce norepinephrine, a neurotransmitter involved in synaptic transduction (Fernstrom and Fernstrom 2007). Depletion of norepinephrine reduces LTP in dentate gyrus of rat hippocampus (Stanton and Sarvey 1985). Further research is merited to unravel the relationships between phenylalanine, norepinephrine, effects on hippocampus, and LTP.

To determine individual stereoisomer contribution to the commercial mixture effects observed in the literature, ~ 10% alpha (3 mg/kg) and 90% gamma (30 mg/kg) metabolic profiles were compared to the 30 mg/kg CM-HBCD. Of the 15 metabolites differentiating CM-HBCD from the two other treatment-groups, all 15 metabolites are seen to be contributed by either α-HBCD or γ-HBCD, with unique profiles between stereoisomers (Figure 5). This supports the hypothesis that α-HBCD and γ-HBCD individually represent a fraction of CM-HBCD’s response, and both individual stereoisomers elicit specific and unique metabolite effects that differ from one another and with respect to CM-HBCD.

The α-HBCD metabolomics profile raises additional concerns, as α-HBCD is formed by stereoisomerization, bioaccumulates, and is the main stereoisomer in human serum and breast milk (Covaci et al. 2006). The metabolites specific to α-HBCD differentiation by treatment and dose groups from the controls (not observed after exposure to CM-HBCD) included increases in the endogenous metabolites acetoacetate, leucine, and tyrosine and decreases in glutamate, lactate, pyruvate, and serine (Figure 5). Current national risk assessments conducted by the EU, Canada, and Australia used CM-HBCD research investigations to derive regulatory risk values (Aylward and Hays 2011). Based on differences between stereoisomer toxicokinetics, molecular pathway identification, and serum metabolomics responses reported here, we recommend further HBCD stereoisomer-specific studies and generation of α-HBCD-specific human health risk assessment for public health protection.

Since metabolites represent end products of the genome and proteome, metabolomics holds promise of providing an integrated physiological phenotype (Merrick et al. 2011). Combining metabolomics with other omic techniques may contribute to an understanding of biological responses to xenobiotics. However, due to its relative infancy compared with conventional toxicity assays and other omics, metabolomics conducted on environmental chemicals is sparse. With appropriate study design, metabolomics represents an opportunity to better understand the toxicity of environmental chemicals and their impact on human health.

In summary, we investigated the toxicity of CM-HBCD, α-HBCD, and γ-HBCD in neonatal mice using a metabolomics profiling approach. All treatment groups exhibited changes in metabolites involved in aerobic energy metabolism, lipolysis, neurodevelopment, and amino acid metabolism. However, each treatment group clustered and separated from the others, signifying different biological responses to each stereoisomer and the mixture. We demonstrate that metabolomics is a promising approach to uncover differences between individual stereoisomers and their mixture, identify potential biomarkers of effects, and discover potential mechanistic links between HBCD exposures and disease and dysfunction.

## Supplemental Material

(151 KB) PDF
