# Supplementary material for: Serum Metabolomic Profiles in Neonatal Mice following Oral Brominated Flame Retardant Exposures to Hexabromocyclododecane (HBCD) Alpha, Gamma, and Commercial Mixture
Source: Environ Health Perspect. 2016 Nov 4;125(4):651–9. doi: 10.1289/EHP242 (PMC5381977; doi:10.1289/EHP242)
Supplement: (151 KB) PDF [file EHP242.s001.acco.pdf]

Environ Health Perspect
DOI: 10.1289/EHP242

Note to readers with disabilities: *EHP* strives to ensure that all journal content is accessible to all readers. However, some figures and Supplemental Material published in *EHP* articles may not conform to 508 standards due to the complexity of the information being presented. If you need assistance accessing journal content, please contact ehponline@niehs.nih.gov. Our staff will work with you to assess and meet your accessibility needs within 3 working days.

Supplemental Material

Serum Metabolomic Profiles in Neonatal Mice following Oral Brominated Flame Retardant Exposures to Hexabromocyclododecane (HBCD) Alpha, Gamma, and Commercial Mixture

David T. Szabo, Wimal Pathmasiri, Susan Sumner, and Linda S. Birnbaum

Table of Contents

Table S1. Mean (SD) concentrations of 40 endogenous metabolites and formate (internal control) with SD according to treatment group.

Table S1. Mean (SD) concentrations of 40 endogenous metabolites and formate (internal control) with SD according to treatment group.

	Control	Commercial	α-HBCD			γ-HBCD	
**Metabolite**	**0 mg/kg n = 6**	**30 mg/kg n = 6**	**3 mg/kg n = 3**	**10 mg/kg n = 3**	**30 mg/kg n = 6**	**3 mg/kg n = 3**	**30 mg/kg n = 6**
**2-HYDROXYISOBUTYRATE**							
Mean	0.0060	0.0062	0.0064	0.0071	0.0070	0.0063	0.0076
SD	0.0003	0.0009	0.0019	0.0014	0.0009	0.0015	0.0011
**3-HYDROXYBUTYRATE**							
Mean	0.1688	0.1921	0.1766	0.2286	0.1781	0.2148	0.2028
SD	0.0153	0.0219	0.0240	0.0402	0.0227	0.0416	0.0248
**ACETATE**							
Mean	0.0086	0.0103	0.0074	0.0083	0.0086	0.0069	0.0081
SD	0.0009	0.0020	0.0013	0.0015	0.0006	0.0012	0.0010
**ACETOACETATE**							
Mean	0.0169	0.0157	0.0178	0.0282*	0.0209	0.0178	0.0180
SD	0.0021	0.0020	0.0024	0.0028	0.0044	0.0049	0.0019
**ACETONE**							
Mean	0.0046	0.0047	0.0045	0.0055	0.0036**	0.0048	0.0046
SD	0.0003	0.0006	0.0006	0.0009	0.0001	0.0003	0.0003
**ALANINE**							
Mean	0.0921	0.0856	0.0723	0.0862	0.0832	0.0810	0.0913
SD	0.0079	0.0057	0.0089	0.0089	0.0040	0.0058	0.0047
**ARGININE**							
Mean	0.0998	0.0912	0.0769**	0.0948	0.0983	0.0674**	0.0974
SD	0.0056	0.0145	0.0054	0.0084	0.0120	0.0025	0.0102
**ASPARAGINE**							
Mean	0.0197	0.0244	0.0174	0.0218	0.0202	0.0158	0.0247
SD	0.0010	0.0034	0.0019	0.0026	0.0025	0.0022	0.0026
**CHOLINE**							
Mean	0.0163	0.0190	0.0154	0.0169	0.0114**	0.0170	0.0146
SD	0.0009	0.0025	0.0012	0.0007	0.0009	0.0003	0.0005
**CITRATE**							
Mean	0.0301	0.0337	0.0277	0.0299	0.0271	0.0488	0.0356
SD	0.0017	0.0024	0.0021	0.0007	0.0012	0.0187	0.0026
**CREATINE**							
Mean	0.0795	0.0847	0.0861	0.0861	0.0829	0.1324	0.0842
SD	0.0034	0.0067	0.0147	0.0003	0.0026	0.0536	0.0054
**CYSTEINE**							
Mean	0.0309	0.0361	0.0269	0.0269	0.0292	0.0269	0.0293
SD	0.0021	0.0063	0.0000	0.0000	0.0024	0.0000	0.0015
**DIMETHYLAMINE**							
Mean	0.0022	0.0027	0.0021	0.0026	0.0021	0.0039	0.0023
SD	0.0001	0.0004	0.0001	0.0002	0.0002	0.0018	0.0001
**FORMATE**							
Mean	1.0346	1.0372	1.0372	1.0372	1.0372	1.0372	1.0372
SD	0.0026	0.0000	0.0000	0.0000	0.0000	0.0000	0.0000
**GLUCOSE**							
Mean	0.9253	0.8910	0.9298	0.9267	0.8986	0.9149	1.0007
SD	0.0703	0.0604	0.0763	0.0164	0.0295	0.0931	0.0963
**GLUTAMATE**							
Mean	0.0669	0.0701	0.0585	0.0560**	0.0523**	0.0716	0.0565
SD	0.0046	0.0067	0.0048	0.0052	0.0010	0.0053	0.0035
**GLUTAMINE**							
Mean	0.0803	0.0871	0.0786	0.0877	0.0834	0.0835	0.0899

	Control	Commercial	α-HBCD			γ-HBCD	
**Metabolite**	**0 mg/kg n = 6**	**30 mg/kg n = 6**	**3 mg/kg n = 3**	**10 mg/kg n = 3**	**30 mg/kg n = 6**	**3 mg/kg n = 3**	**30 mg/kg n = 6**
SD	0.0036	0.0074	0.0036	0.0049	0.0028	0.0024	0.0046
**GLYCEROL**							
Mean	0.1004	0.1225*	0.0908	0.1085	0.0979	0.1257	0.1114
SD	0.0058	0.0048	0.0095	0.0138	0.0048	0.0138	0.0092
**GLYCINE**							
Mean	0.0880	0.0910	0.0825	0.0985	0.0911	0.1017	0.1024
SD	0.0044	0.0058	0.0106	0.0075	0.0095	0.0066	0.0061
**HOMOSERINE**							
Mean	0.1677	0.1591	0.1591	0.1591	0.1591	0.1591	0.1591
SD	0.0086	0.0000	0.0000	0.0000	0.0000	0.0000	0.0000
**ISOBUTYRATE**							
Mean	0.0046	0.0046	0.0039	0.0037	0.0044	0.0040	0.0041
SD	0.0002	0.0004	0.0005	0.0006	0.0002	0.0005	0.0003
**ISOLEUCINE**							
Mean	0.0220	0.0238	0.0204	0.0257	0.0237	0.0230	0.0222
SD	0.0019	0.0029	0.0030	0.0021	0.0022	0.0020	0.0015
**LACTATE**							
Mean	0.9056	1.0190	0.7690	0.6941	0.7012	0.8190	0.8231
SD	0.0745	0.0752	0.0780	0.1154	0.0472	0.0451	0.0590
**LEUCINE**							
Mean	0.0354	0.0361	0.0394	0.0408	0.0395	0.0372	0.0389
SD	0.0028	0.0040	0.0085	0.0022	0.0033	0.0055	0.0022
**N LYSINE**							
Mean	0.0509	0.0549	0.0549	0.0496	0.0502	0.0491	0.0464
**SD**	0.0053	0.0082	0.0036	0.0021	0.0041	0.0088	0.0041
**METHANOL**							
Mean	0.0101	0.0055	0.0241	0.0049	0.0050	0.0057	0.0067
SD	0.0033	0.0009	0.0106	0.0007	0.0009	0.0012	0.0015
**METHIONINE**							
Mean	0.0252	0.0296	0.0256	0.0310	0.0245	0.0274	0.0259
SD	0.0021	0.0041	0.0003	0.0033	0.0011	0.0014	0.0027
**METHYLSUCCINATE**							
Mean	0.0205	0.0191	0.0192	0.0231	0.0214	0.0204	0.0191
SD	0.0030	0.0018	0.0036	0.0021	0.0025	0.0040	0.0018
**N,N-DIMETHYLGLYCINE**							
Mean	0.0052	0.0044	0.0043	0.0045	0.0041	0.0058	0.0046
SD	0.0005	0.0003	0.0005	0.0008	0.0003	0.0017	0.0004
**O-PHOSPHOCHOLINE**							
Mean	0.0375	0.0478	0.0369	0.0527*	0.0371	0.0370	0.0343
SD	0.0039	0.0052	0.0032	0.0056	0.0022	0.0062	0.0034
**PHENYLALANINE**							
Mean	0.0204	0.0168	0.0205	0.0198	0.0177	0.0150**	0.0144**
SD	0.0013	0.0014	0.0024	0.0014	0.0010	0.0007	0.0018
**PYRUVATE**							
Mean	0.0447	0.0447	0.0407	0.0394	0.0321*	0.0413	0.0398
SD	0.0019	0.0040	0.0030	0.0040	0.0024	0.0001	0.0011
**SARCOSINE**							
Mean	0.0120	0.0126	0.0118	0.0121	0.0119	0.0179	0.0121
SD	0.0001	0.0005	0.0002	0.0002	0.0001	0.0063	0.0002
**SERINE**							
Mean	0.0623	0.0628	0.0446	0.0665	0.0597	0.0772	0.0526
SD	0.0061	0.0065	0.0056	0.0072	0.0030	0.0139	0.0037
**SUCCINATE**							
Mean	0.0148	0.0153	0.0109	0.0092	0.0115	0.0128	0.0141

Metabolite	Control	Commercial	α-HBCD			γ-HBCD	
	0 mg/kg n = 6	30 mg/kg n = 6	3 mg/kg n = 3	10 mg/kg n = 3	30 mg/kg n = 6	3 mg/kg n = 3	30 mg/kg n = 6
SD	0.0021	0.0019	0.0014	0.0012	0.0011	0.0020	0.0010
**TAURINE**							
Mean	0.1697	0.1913*	0.1769	0.1854	0.1691	0.2440	0.1825
SD	0.0055	0.0012	0.0128	0.0043	0.0075	0.0823	0.0091
**THREONINE**							
Mean	0.0665	0.0700	0.0459	0.0635	0.0543	0.0634	0.0525
SD	0.0103	0.0139	0.0037	0.0064	0.0064	0.0085	0.0065
**TYROSINE**							
Mean	0.0324	0.0294	0.0354	0.0373	0.0310	0.0328	0.0278
SD	0.0042	0.0021	0.0024	0.0005	0.0020	0.0029	0.0033
**VALINE**							
Mean	0.0258	0.0298	0.0262	0.0315	0.0320	0.0268	0.0283
SD	0.0022	0.0028	0.0030	0.0007	0.0030	0.0038	0.0015
**MYO-INOSITOL**							
Mean	0.0278	0.0300	0.0172	0.0190	0.0227	0.0369	0.0242
SD	0.0035	0.0043	0.0051	0.0021	0.0023	0.0173	0.0034
**METHYLHISTIDINE**							
Mean	0.0200	0.0203	0.0168	0.0199	0.0196	0.0185	0.0196
SD	0.0015	0.0033	0.0015	0.0004	0.0012	0.0012	0.0005

*Significantly higher than control, $p < 0.05$

** Significantly lower than control, $p < 0.05$
